# Biobank integration of large-scale clinical and histopathology melanoma studies within the European Cancer Moonshot Lund Center

**DOI:** 10.1186/s40169-018-0203-8

**Published:** 2018-08-20

**Authors:** Johan Malm, Yutaka Sugihara, Marcell Szasz, Ho Jeong Kwon, Henrik Lindberg, Roger Appelqvist, György Marko-Varga

**Affiliations:** 1 Department of Translational Medicine, Section for Clinical Chemistry, Lund University, Skåne University Hospital Malmö, 205 02 Malmö, Sweden; 2 Clinical Protein Science & Imaging, Biomedical Center, Dept. of Biomedical Engineering, Lund University, BMC C13, 221 84 Lund, Sweden; 3 Centre of Excellence in Biological and Medical Mass Spectrometry, Lund University, BioMedical Centre D13, 221 85 Lund, Sweden; 4 Department of Oncology, Clinical Sciences, Lund University, Skåne University Hospital, 221 85 Lund, Sweden; 5 2nd Department of Pathology, Semmelweis University, Budapest, 1091 Hungary; 6 Chemical Genomics Global Research Lab, Department of Biotechnology, College of Life Science and Biotechnology, Yonsei University, Seoul, 120-749 Republic of Korea

## Abstract

We present the Cancer Moonshot clinical project located at the European center in Lund. Here, tissue and blood samples have been collected and stored in a large-scale biobank. Multiple clinical centers around the world are participating and tissue and blood samples are sent to the European Cancer Moonshot Lund Center that acts as the clinical hub. Our center has been developed to generate and build large-scale biostorage archives of patient melanoma samples, which is then combined with a histopathological capability to characterize the patient tumours. Such a large-scale clinical sample processing initiative has begun with the aim of creating high-end histopathology indexing with database computational power and including proteogenomic analysis. The biobank at Lund has become an important resource in clinical research worldwide. Following suite, several national health programs are being initiated with the aim of also building large-scale biobank storages with a wealth of high-quality patient samples. In our Cancer Moonshot R&D activities, samples in the biobanks and the data derived from these samples are being used to build an understanding of disease presentation and using this information to move towards ‘Big Data’ proteogenomic and mass spectrometry imaging studies. Additionally, we report here a sample processing workflow that has been adapted to a fully-automated biobank processing strategy for large-scale studies.

## Background

Today, cancer impact by optimal treatment is already at the front line worldwide with the latest immuno- and cell therapies. To reach many of these milestone achievements, researchers have been storing hundreds of millions of specimens in various biobanks. Recent reports have indicated that the rate of sample collection is in the order of 20 million samples per year [1, 2]. In Europe, the council of the European Union has approved the policy on human biological specimens in biobanks. By relating the number of biobank samples to the number of cancer patients, there is an estimate of 14,738,719 people living with cancer of any type in the United States (2014). Based on 2012–2014 data, it is predicted that approximately 38.5% of men and women will be diagnosed with cancer of any type at some point during their lifetime [3]. By 2020, the estimated cost for cancer care in the US alone is estimated to be $158 billion. This value was predicted by the NIH, where changes within the US population and forthcoming cancer trends were taken into consideration.

In Sweden, a relatively small country (population approx. 10.1 Mill.), approximately 60.000 new cancer diagnoses are made every year and every third person will be diagnosed with cancer at some time during their life time. The incidence of cancer has been increasing for many years and the total annual cost of cancer care has been estimated to more than four billion euro. Every year 3–4 Million new tissue samples (incl. blood) are added to the existing + 150 Mill. samples.

Amongst all cancers, malignant melanoma is the fifth most common cancer type. In 2017, there was an estimated increase of 87,110 new cases, and an estimated 9730 deaths. As such, melanoma of the skin represents 5.2% of all new cancers in the US only [4]. For comparison, the annual number of new melanoma cases in Sweden are approx. 5000 and the incidence is now more than four times higher than 25 years ago. Globally, there is an increasing interest and need to support research areas that can help solve disease understanding and improve patient care. This includes approaches such as ‘precision medicine’, alternative treatment technologies and early indication of disease diagnosis utilizing both imaging techniques and biomarker diagnostics [5, 6]. Ultimately, it is the patients who suffer and experience the current limitations in treatment. From an international perspective, the biobanking developments within the Cancer Moonshot activities in Lund have thus been given top priority to provide high-quality and well-defined tissue and biofluid specimens [7–9]. The center includes pathological characterization, where the tumour tissues are evaluated with high integrity and in great detail, with annotations that relate to the types of cells presented in the tumour tissues, their nature, and the morphology captured by histology images. Associated clinical data are also included into the RedCap database. In real time, the mapping and monitoring of the study progress occurs with our clinical partners worldwide [10, 11].

## Malignant melanoma

Malignant melanoma is the most dangerous type of skin cancer, and develops from pigment-containing cells known as melanocytes. These cells occur primarily in the skin, however, such cells also exist on the genitalia, in the mouth and the eye. Additionally, men often have a higher occurrence on the back; whilst with women, the most common occurrence is on the legs. It is known that the primary cause of melanoma is exposure to UV light. This risk increases when combined with low levels of skin pigment, a compromised immune system, and other genetic factors [3, 4].

Most commonly, the diagnosis of a melanoma is determined from any suspicious skin lesion. Summarizing the basic melanoma pathology, patients are grouped into: superficial spreading melanoma (SSM), nodular melanoma (NM), lentigo maligna melanoma (LMM) and acral lentiginous melanoma (ALM) sub-groups, as presented in Table 1.

**Table 1:** Melanoma Pathology (Proposed by Mool and Krausz)

	SSM	NM	LMM	ALM
Peak incidence	Middle adult life	Middle adult life	Late adult life	Late adult life
Race	Usually whites	Usually whites	Only whites	All races
Localization	All skin	All skin	Sun-damaged skin	Palms, Soles, Subungual
Main cell type in epidermis	Epithelioid	Epithelioid	Dendritic	Dendritic
Pagetoid lateral spread	Usual	Absent and minimal	Rare	Rare
Lentiginous lateral spread	Uncommon	Absent	Usual	Usual
Epidermal hyperplasia	Often	Sometimes	Absent	Present
Epidermal atrophy	Sometimes	Sometimes	Present	Absent
Actinic damage of skin	Sometimes	Sometimes	Present	Absent
Relative frequencies (Clark et al. [12]) unclassifiable: 10%	67%	10%	9%	4%

**Fig. 1:**
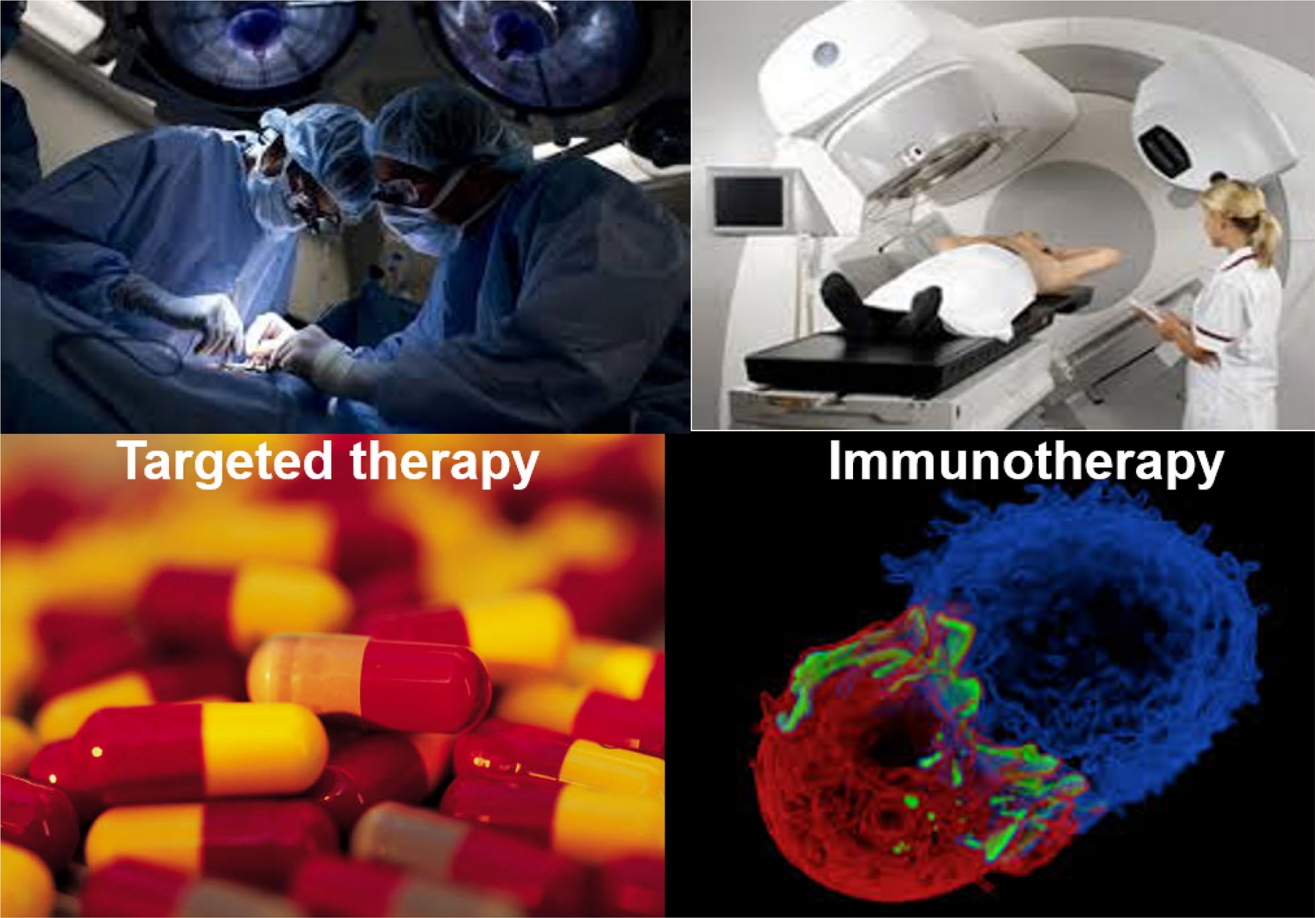
Several types of treatment are used for melanoma patients; surgery, radiation, drug therapy, and immunotherapy. These treatments are often used in combination—especially for patients with metastatic disease

The usual treatments for these patient groups are presented in Fig. 1. Here, surgery is still perhaps the most pre-emptive and efficient method with a good outcome for the patient. As melanoma tumours usually intensively metastasize throughout the body, it can be challenging to manage with surgical intervention alone. This is particularly the case if the tumour has metastasized to multiple organs and sites. When this occurs, alternative treatment is usually necessary. These include chemotherapy, traditional, or targeted treatments using precision or personalised medicine; and can be combined with or without radiation (see Fig. 1).

Typically, surgical removal of a tumour is the most efficient approach. Assessment of nearby lymph nodes for metastases is also often performed; and in general, most people do not relapse if metastases has not occurred. With treatment, the 5-year survival rate in the United States for those with a localised disease, and metastatic spread, is 98 and 17%, respectively. The likelihood of re-occurrence or metastases is dependent on the thickness of the original tumour and/or how rapidly the cells are dividing.

The new generation of omic-integrative medicine includes genomic and proteomic expression data analysis in the final diagnosis and decision on the best treatment for patients. Genomic changes, however, are not always present at the protein level. This suggests that the additional level of proteomic analysis will lead to an enhanced functional understanding of tumour resistance and/or toxicity to therapy. Ultimately, this may lead to the ability to predict treatment response by examining drug response or toxicity. One of the major objectives of the Cancer Moonshot program is the belief that integrating genomic and proteomic data can provide more information and insight into the development and growth of cancer; and the most efficient treatment, preferably with precision medicine.

**Fig. 2:**
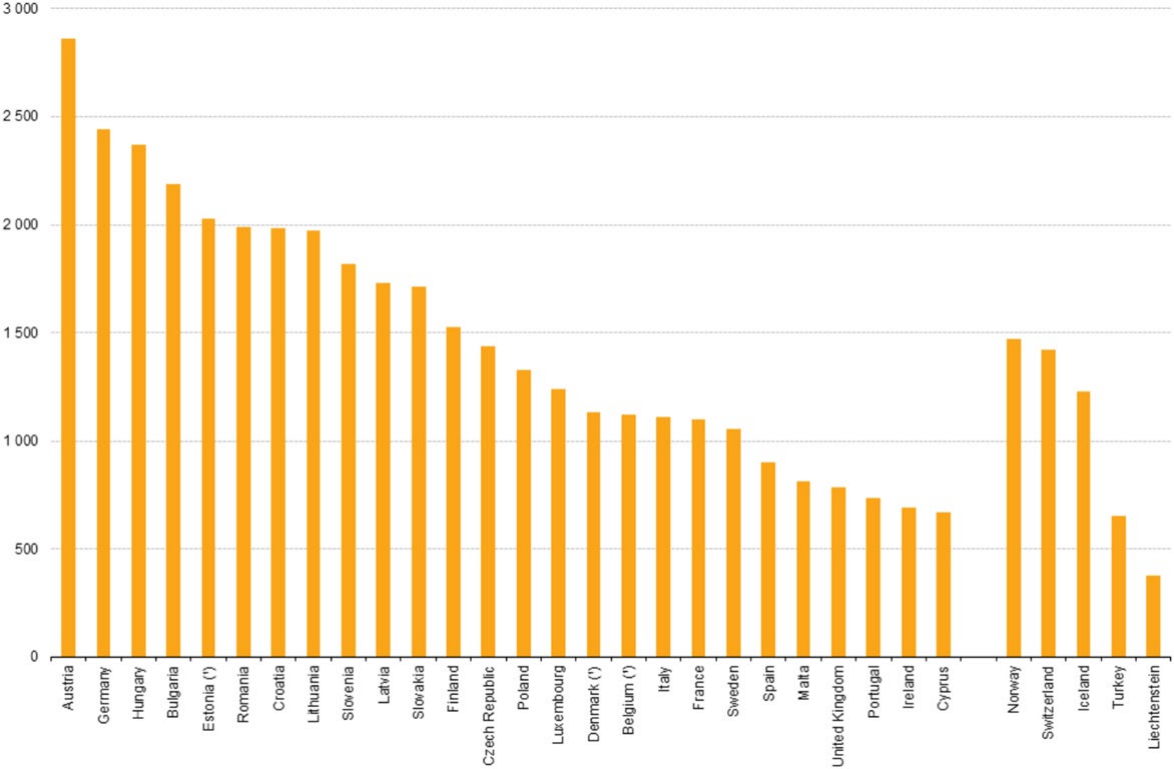
Hospital discharge rates for in-patients with neoplasms, 2014 (per 100,000 inhabitants). Note the substantial variation between different European countries (source: Eurostat)

Considering the ever-increasing number of cancer patients worldwide, we are facing a real challenge in society both in terms of healthcare efficiency and scientific delivery of new principles. Particularly for the latter, an understanding of the mechanisms of cancer development is imperative both in terms of drug treatment and disease diagnosis. An overview of European hospital discharge rates for in-patients with neoplasms is depicted in Fig. 2. A substantial variation is seen with the highest rates in Austria, Germany and Hungary.

## Biobank impact on cancer healthcare

To build clinical value in the future by combining patient care and expanding large-scale sample collections, there are three major groups that need to work together. Overall healthcare costs are increasing and according to the Centers for Medicare and Medicaid Services, healthcare expenditure accounted for 17.8% of the U.S. gross domestic product in 2015. There are a variety of factors that contribute to the increased cost and varying opinions on how resources should be allocated to account for the cost gap. One thing is certain: the United States spends an inordinate amount of money on healthcare. The strain on the economy is obvious, and healthcare professionals are attempting new and innovative measures to contain costs [11, 13].

Recently, there was a report on the economic aspects of biobanks, particularly those facilitated by the state [14]. It has been noted that national biobanks are often funded by public/private partnerships, with finance provided by a combination of national research councils, medical charities, pharmaceutical company investment and biotech venture capital [15]. In this way, national biobanks enable an economic relationship mediated between states, national populations, and commercial entities. It has been illustrated that there is a strong commercial incentive underlying the systematic collection of tissue material. This is particularly evident in the field of genomic research, where population-sized studies lend themselves more easily towards diagnostic technologies rather than basic etiological studies [16].

Also legal aspects of biobanking patient samples have to be considered, not only for establishing the physical biobank but also for each study that uses the biobank for storage. The Lund biobank has been designed according to Swedish law and recommendations and for each study ethical approval by the regional ethical committee is required—both for storing samples and for using samples stored in the biobank.

**Fig. 3:**
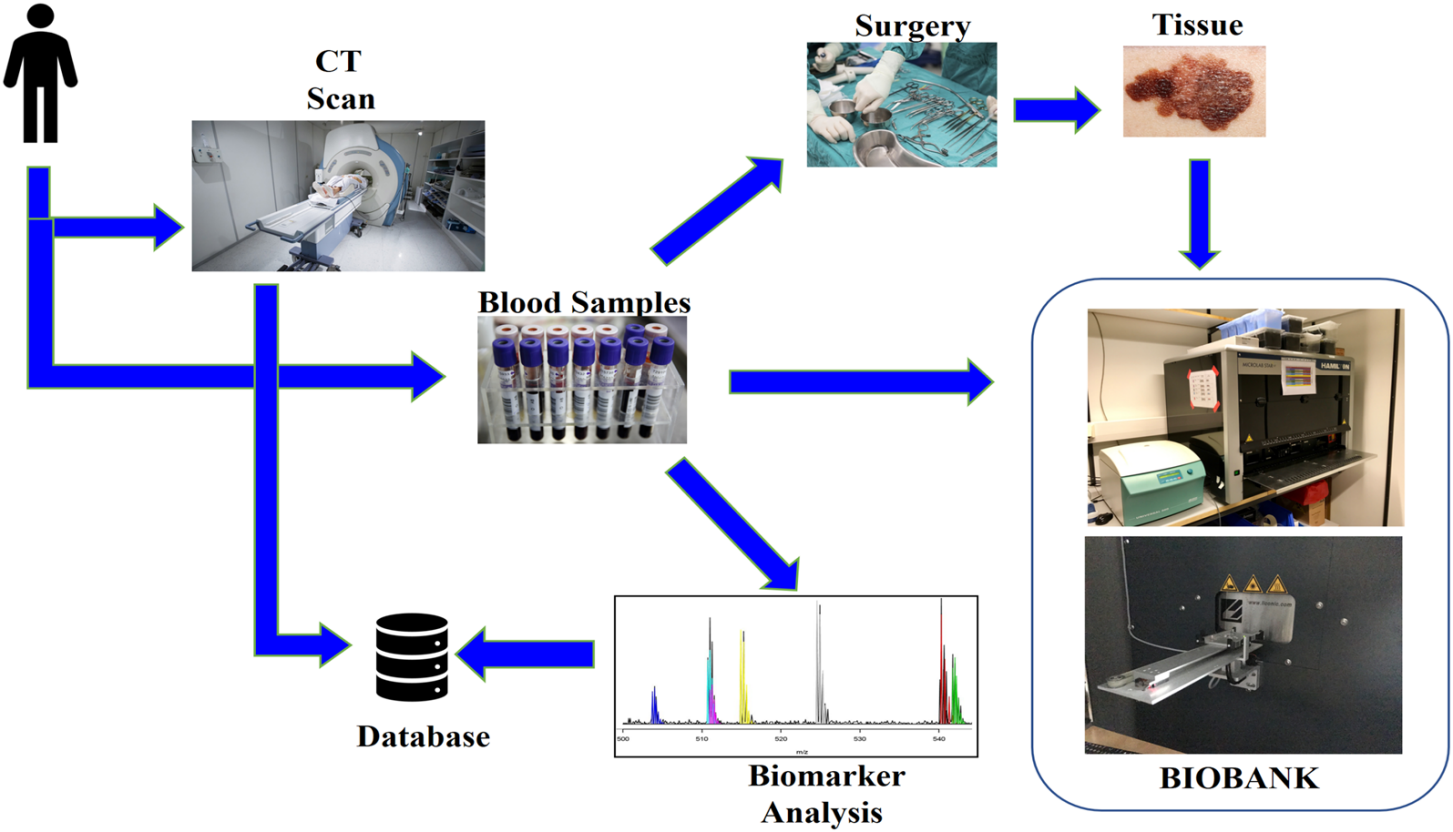
Schematic illustration of the biobank workflow that has been developed and implemented in Lund. Blood and tissue samples (primary tumour and metastases) are stored in the automated biobank and can be used in research projects, e.g. aiming at finding new biomarkers

**Fig. 4:**
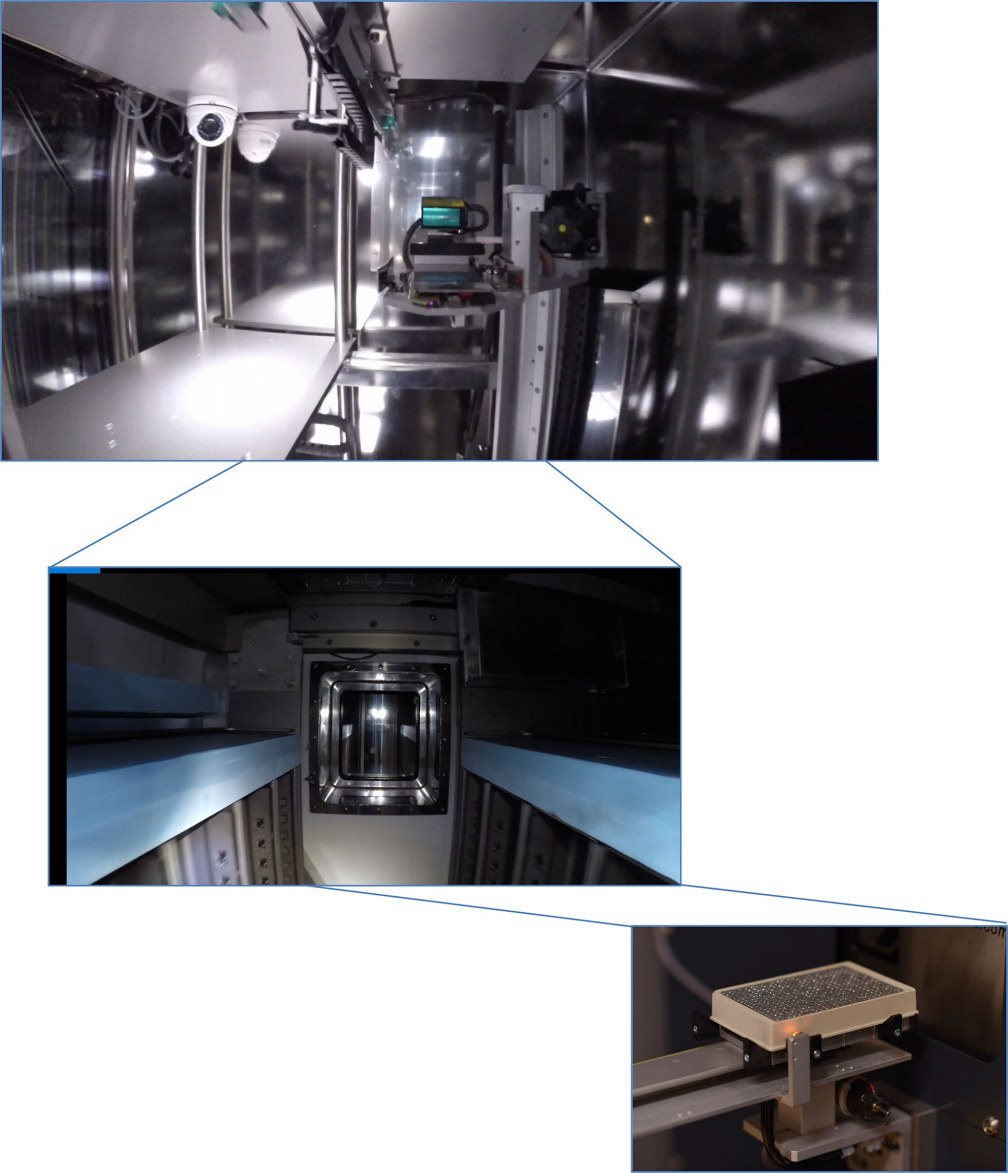
The fully-automated robotics of the biobank is shown; the heart of the robotic entry point is shown in the upper image; whilst the upper view of the robot is given in the central image. The lower image shows the automated biobank storage with the robotic 384-sample tube plate entering the − 80 °C compartment

In 2017 at the Lund biobank, approximately 300,000 sample tubes of blood fractions have been processed. Using a dedicated protocol and workflow, most of these were processed within a maximum of 2 h. The patient blood sample tubes samples were then transported to the BMC biobank facility. Here, the blood samples were fractionated by automated liquid handling using the Hamilton work station (see Figs. 3 and 4). When surgery was performed, the tissue samples were processed and stored in the same way as the blood fractions in the automated biobank. The biobank stores all samples with dedicated barcodes and the accompanying data set on the server.

The Cancer Moonshot biobank logistics and program has been outline for the operations in Lund that has been developed to generate and build large-scale biostorage archives of melanoma patient samples for proteogenomic analysis. Within Region Skåne, the Southern Swedish healthcare, within the melanoma patient treatment, we are following the progression and treatment of each respective patient. In practice, this means that whenever a melanoma patient enters the hospital in Lund or other hospitals in the region, sampling and clinical follow up is being made that our study can follow up. In most cases, blood can be taken from the patients. If surgical intervention is required, part of the patient tissue is routinely linked into the workflow we have built. The team work between our proteomic unit and the clinicians at the hospital is very good. Senior scientists, post-doctoral fellows and Ph.D. students are trained, and moved into this important biobanking activity.

The integrity of the biobank is of key importance, as the benefiters will be provided with high quality clinical material and associated clinical data.

The three major stakeholder groups that are involved in the daily biobank operations are:

*Patients* that contribute by sample donation, where proteogenomic profiling information concerning the tumour is undertaken. Given the genomic profile of the tumour combined with other relevant information, such as clinical data, it will be possible to learn which treatments may provide the best outcome for the patient.

*Researchers* can identify possible targets for the development of new treatments and preventive interventions. This will include early indication of disease onset, determining how to optimally treat a patient, and how to avoid or counter drug resistance. Access to samples stored in the biobank and associated clinical information can be given in collaborative research projects with the European Cancer Moonshot Lund team.

*Medical doctors* will have access to information that predicts better treatment outcomes and helps control the symptoms and side effects in the patient. The earlier cancer treatment is initiated, the better the outcome for the patient [17–19].

## Characterising cancer tissue heterogeneity

Currently, there are a number of clinical studies that show that tumour heterogeneity is linked to the survival of cancer patients, i.e., lower heterogeneity correlates with longer survival. Throughout 2017 in a collaboration study with the Semmelweis Oncological Dermatology team in Budapest, Hungary, surgically-isolated melanoma tumours have been processed. A workflow to handle and process surgically-removed tumour tissue has been built.

Once the histopathological evaluation was performed, the areas of the different tissue compartments were manually outlined to create a mask plane for each cell type. These mask planes were then used to query specific mass spectrometry-generated ion signatures that are correlated or anti-correlated with a given histological structure or cell type.

By mapping the compartments within the tissue by imaging mass spectrometry, the sections of the tumours were divided into: (i) melanoma cells; (ii) lymphocytes; and (iii) macrophages.

**Fig. 5:**
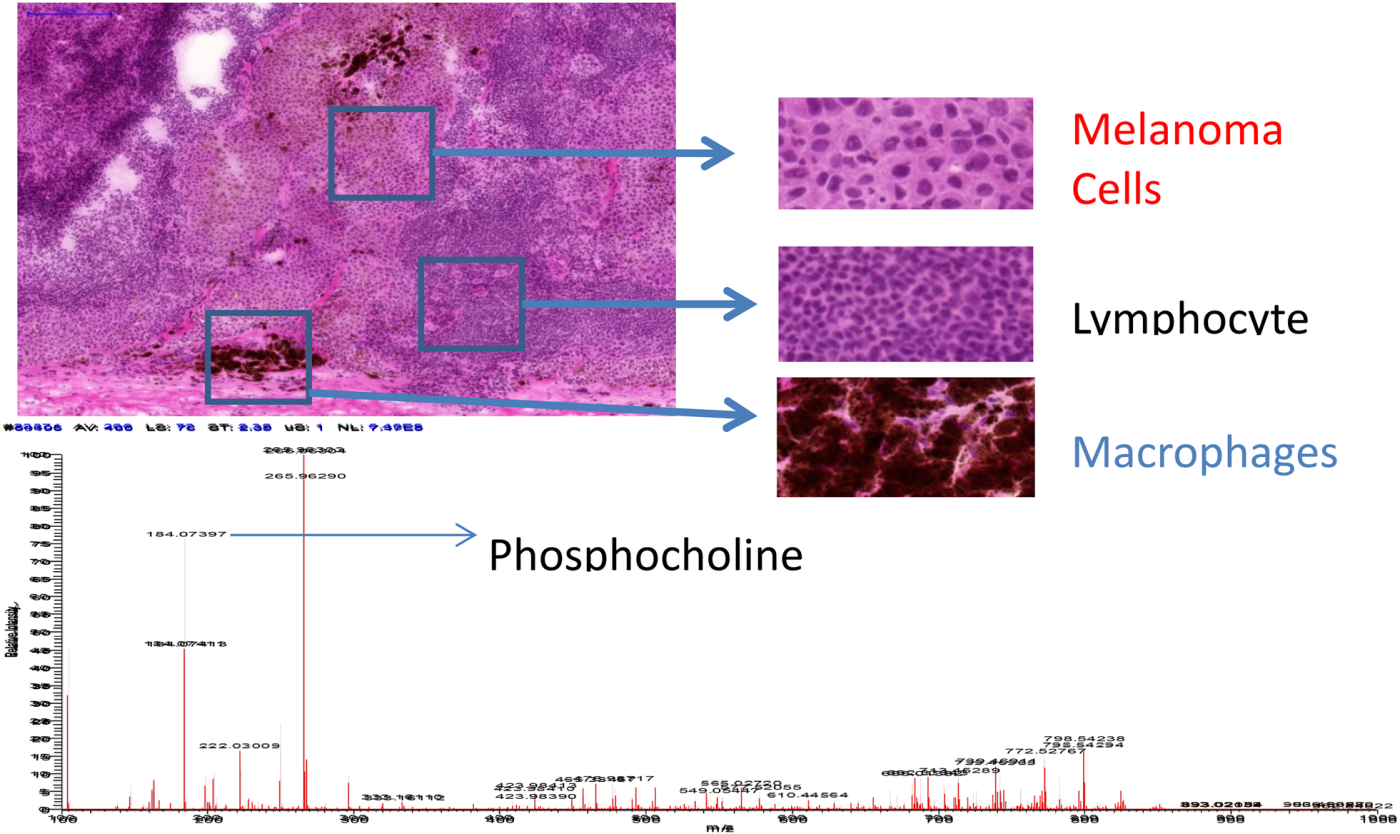
Tissue compartment presentation in a melanoma tissue sample—different cell types dominate in different parts of the section. The corresponding mass spectrum is also given (lower panel). The coverage over the tumour was shown to vary substantially. Entire coverage was apparent for metabolites with *m/z* values of 184.073 and 222.030; whilst the metabolite at *m/*z 296 only showed expression in the upper epithelial part of the tumour (data not shown). Phosphocholine is used as a reference

Figure 5 illustrates the heterogeneity of the tumour with different cell types dominating in different compartments of a melanoma tissue sample. The corresponding mass spectrum is also given (lower panel). The coverage over the tumour was shown to vary substantially. Entire coverage was apparent for metabolites with *m/z* values of 184.073 and 222.030; whilst the metabolite at *m/z* 296 only showed expression in the upper epithelial part of the tumour (data not shown).

**Fig. 6:**
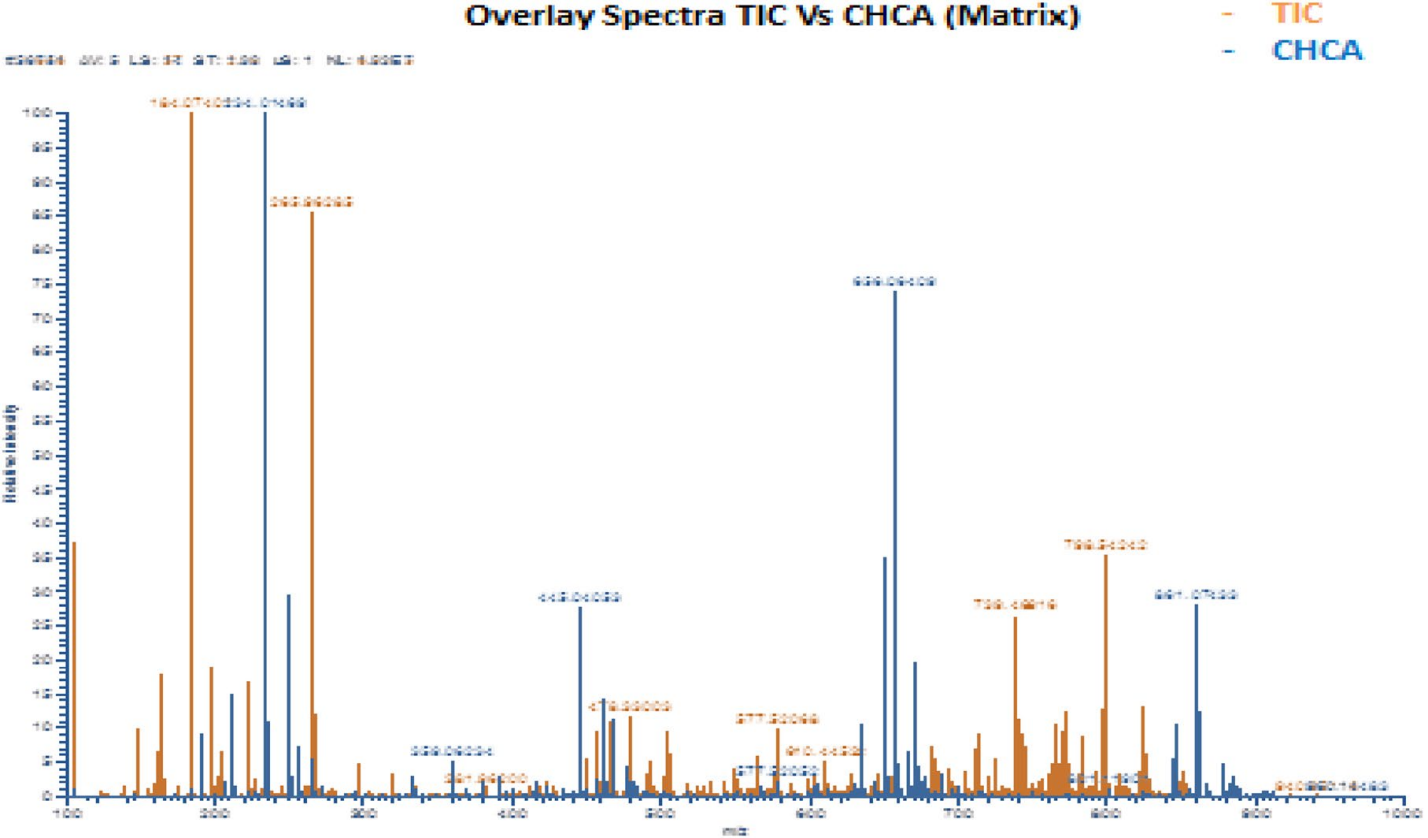
Resultant mass spectrum from a melanoma tumour. TIC—total ion current, CHCA—α-cyano-4-hydroxycinnamic acid (matrix). All metabolites are shown as singly- or doubly-charged ions within the tumour tissue

A corresponding mass spectrum is depicted in Fig. 6, where all metabolites are shown as singly- or doubly-charged ions within the tumour tissue. Depending on the instrumentation used, the technique also offers high spatial resolution (to the cellular level). At present MSI is performed only on selected cases to demonstrate tumour heterogeneity. Different ionisation methods are available, but matrix-assisted laser desorption/ionisation (MALDI) is perhaps the most widespread in mass spectrometry imaging (MSI). In MALDI, a so-called matrix compound is applied to the samples. The matrix absorbs the energy from the laser which is transferred to the analyte via a process referred to as ‘soft’ ionisation. MALDI-MSI is widely-used to characterise drug distribution in various tissue types. The method is also used to investigate various endogenous molecules, such as lipids, carbohydrates, peptides and proteins. MSI has gained significant interest over the past few decades from the pharmaceutical community. As a result of continued technical development, MSI will undoubtedly become increasingly important in pathology and in the clinic.

After H&E staining, the same tissue section was histologically characterized. The pathologists identified several tissue compartments and cell types in the analysed tissue samples, i.e., cancer cells, lymphocytes, macrophages, connective tissue and other tissue compartments. The H&E stained image section of a melanoma proliferative lesion and attached subcutaneous tissue is shown in the images above. This is a representative illustration of a patient tumour sample that was processed and analysed within the European Cancer Moonshot Center Lund laboratories.

The H&E stained section showed a melanoma proliferative lesion with attached subcutaneous tissue. Dermal involvement of atypical melanocytes with cytologic atypia was observed. The tumour lesion was divided into two areas by morphological features of melanocytes. One area contained large melanocytes with abundant cytoplasm and polygonal nuclei (Fig. 5, melanoma cells). Additionally, another area contained small melanocytes with poor cytoplasm and small nuclei composed of dense chromatin (Fig. 5, lymphocyte). Also, an infiltration area of brown pigment-laden macrophages was observed in the tumour proliferative lesion and attached subcutaneous tissue (Fig. 5, macrophages).

**Fig. 7:**
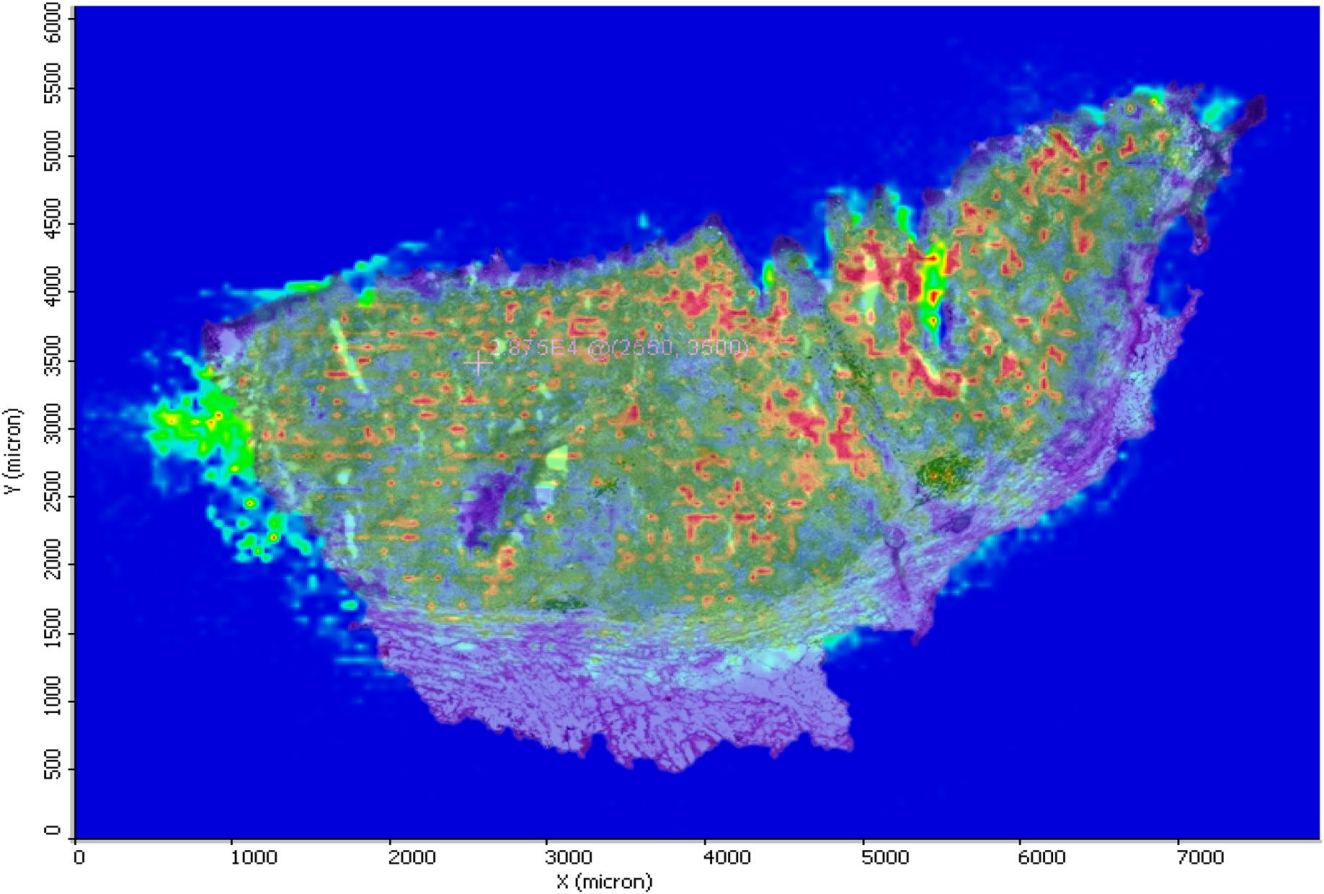
MALDI mass spectrometry image overlay with histology from a patient cancer tissue sample isolated after surgery demonstrating tumour heterogeneity and metabolite localization. Spatial resolution indicated

MALDI MSI demonstrated specific ions from each area. In addition, what is reported here reflects that the vast majority of the signal defined as a metabolite is clearly located within recognisable structures within the tissue, illustrated in a counterplot image as shown in Fig. 7.

In conclusion, the European Cancer Moonshot Lund Center, an infrastructure that is associated with one of the world’s largest cancer projects, has now been in operation since June 2017 and we hope it will initiate many collaborative studies.

## Abbreviations

MALDI-MSI: matrix-assisted laser desorption ionisation mass spectrometry imaging
MSI: MS imaging
H&E: hematoxylin & eosin
